# Comparative Analysis of Machine Learning Methods with Chaotic AdaBoost and Logistic Mapping for Real-Time Sensor Fusion in Autonomous Vehicles: Enhancing Speed and Acceleration Prediction Under Uncertainty

**DOI:** 10.3390/s25113485

**Published:** 2025-05-31

**Authors:** Mehmet Bilban, Onur İnan

**Affiliations:** 1 Department of Computer Technologies, Necmettin Erbakan University, Konya 42370, Turkey; mbilban@erbakan.edu.tr; 2 Department of Computer Engineering, Faculty of Technology, Selcuk University, Konya 42150, Turkey

**Keywords:** AdaBoost, Apache Kafka, Artificial Neural Networks, autonomous vehicles, Gradient Boosting, k-Nearest Neighbors, machine learning, Random Forest, Chaotic AdaBoost

## Abstract

**Highlights:**

- CAB’s chaos-enhanced ensemble learning sets a new standard for AV sensor fusion, achieving unmatched prediction accuracy.
- The integration of Apache Kafka 2.13-3.4.0 and MongoDB 7.0.4 with CAB ensures real-time robustness, enhancing AV reliability under sensor uncertainties.

**What are the main findings?**

- CAB outperforms traditional methods, offering a scalable solution for speed and acceleration estimation under uncertainty.
- The simulated results demonstrate CAB’s potential, achieving superior safety (TTC: 3.2 s) and comfort (jerk: 0.15 m/s^3^) metrics, though real-world validation is needed.

**What is the implication of the main finding?**

- CAB’s success paves the way for safer, more efficient AV systems, accelerating the adoption of autonomous technologies.
- The need for real-world testing highlights a pathway to refine CAB, potentially establishing it as a cornerstone in AV development beyond simulation constraints.

**Abstract:**

This study presents a novel artificial intelligence-driven architecture for real-time sensor fusion in autonomous vehicles (AVs), leveraging Apache Kafka and MongoDB for synchronous and asynchronous data processing to enhance resilience against sensor failures and dynamic conditions. We introduce Chaotic AdaBoost (CAB), an advanced variant of AdaBoost that integrates a logistic chaotic map into its weight update process, overcoming the limitations of deterministic ensemble methods. CAB is evaluated alongside k-Nearest Neighbors (kNNs), Artificial Neural Networks (ANNs), standard AdaBoost (AB), Gradient Boosting (GBa), and Random Forest (RF) for speed and acceleration prediction using CARLA simulator data. CAB achieves a superior 99.3% accuracy (MSE: 0.018 for acceleration, 0.010 for speed; MAE: 0.020 for acceleration, 0.012 for speed; R^2^: 0.993 for acceleration, 0.997 for speed), a mean Time-To-Collision (TTC) of 3.2 s, and jerk of 0.15 m/s^3^, outperforming AB (98.5%, MSE: 0.15, TTC: 2.8 s, jerk: 0.22 m/s^3^), GB (99.1%), ANN (98.2%), RF (97.5%), and kNN (87.0%). This logistic map-enhanced adaptability, reducing MSE by 88% over AB, ensures robust anomaly detection and data fusion under uncertainty, critical for AV safety and comfort. Despite a 20% increase in training time (72 s vs. 60 s for AB), CAB’s integration with Kafka’s high-throughput streaming maintains real-time efficacy, offering a scalable framework that advances operational reliability and passenger experience in autonomous driving.

## 1. Introduction

AVs are transportation systems that can move safely by sensing their environment and making decisions without human intervention. These vehicles, which work with sensors such as cameras, radar, LIDAR, and artificial intelligence algorithms, are revolutionizing transportation technology today [1]. Autonomous vehicle development efforts began in the 1950s with scientific and military projects and progressed with the emergence of the first prototypes in the 1980s. Competitions such as the DARPA Grand Challenge contributed to the rapid development of this technology in the 2000s, and commercial applications were launched under the leadership of companies such as Google’s Waymo and Tesla [2]. There are different categories in the 5-level autonomy system defined by SAE, from completely driver-controlled to fully autonomous vehicles. Today, autonomous vehicles attract attention, especially with their potential to increase transportation safety and efficiency, and are rapidly becoming widespread with urban test drives and commercial applications. Autonomous vehicles aim to provide a safe and efficient driving experience as one of the most innovative applications of modern transportation technology. These vehicles, which perceive and analyze their environment and act by making independent decisions, create a great revolution with their potential to increase traffic safety and reduce dependency on human intervention. However, the implementation of this technology requires overcoming various technical, ethical, and legal challenges [3,4].

One of the biggest challenges facing AVs is accurately perceiving and making decisions in dynamic, complex environments under sensor uncertainties, such as low light, weather variability, or unexpected obstacles [5], which is compounded by regulatory inconsistencies across regions and ethical dilemmas in crash scenarios [3,4]. Speed and acceleration prediction, critical for safety, is particularly vulnerable to sensor failures, where deterministic ensemble methods like AdaBoost struggle with chaotic data variability. The need for real-time decision making further requires rapid, reliable responses to sudden traffic situations [6,7], while processing large volumes of sensor data remains essential to system performance. Additionally, ethical decision-making processes and cybersecurity threats impact reliability [8,9]. This study introduces CAB, enhancing adaptability via a logistic chaotic map, surpassing conventional approaches in real-time robustness and addressing a key gap in AV reliability.

Machine learning methods play a vital role in addressing these challenges. Machine learning algorithms, in basic processes such as perception, decision making, data analysis, and safety, enable autonomous vehicles to become more reliable and effective. For example, machine learning models are widely used in tasks such as recognizing traffic signs, detecting road lanes, and classifying objects in environmental perception [10]. In addition, these models also provide significant contributions to tasks such as combining data from sensors (sensor fusion) and detecting system anomalies. In real-time decision-making processes, these methods enable vehicles to respond quickly and accurately to sudden situations [11,12].

In order for autonomous vehicles to succeed, the correct application of machine learning methods and the continuous development of these methods are of critical importance [13]. For instance, our previous work [14] utilized Lévy Flight-integrated Proximal Policy Optimization (LFPPO) to optimize autonomous vehicle performance via reinforcement learning, whereas this study employs supervised learning, notably CAB, to enhance regression-based speed and acceleration estimation under sensor uncertainties. While CAB introduces chaotic dynamics to enhance adaptability, other established methods offer distinct strengths: kNN provides simplicity and effectiveness in local pattern recognition, ANN excels in modeling complex non-linear relationships, AB and GB leverage iterative error correction for precision, and RF ensures robustness through diversity and generalization. This study evaluates these methods collectively using simulated data from the CARLA simulator, which, while representative of urban scenarios, imposes controlled conditions that may not fully capture real-world complexities, like variable sensor noise or extreme environmental factors. To address this, we propose future validation with real-world datasets, ensuring practical applicability beyond simulation constraints. This approach highlights the complementary roles of these methods alongside CAB’s innovative chaos-enhanced framework.

Sensor failure in autonomous vehicles is a serious problem that can compromise the safety of critical functions such as acceleration and acceleration control. In such cases, machine learning methods offer an effective solution to ensure the operational reliability of the vehicle [15]. Anomaly detection and failure prediction algorithms can minimize the impact of faulty sensors by identifying errors and omissions in sensor data [16]. Sensor fusion techniques can reconstruct the vehicle’s environmental perception by combining incomplete information from faulty sensors with data from other sensors. In addition, predictive methods such as regression models or neural networks can maintain control of the vehicle by estimating speed and acceleration despite incomplete sensor data. Real-time decision-making algorithms optimize acceleration and braking processes despite faulty sensors, allowing the vehicle to act adaptively. The use of these methods offers significant advantages, such as system flexibility, operational efficiency, and passenger safety, while minimizing safety risks caused by sensor failures. Thanks to machine learning, autonomous vehicles can not only become resilient to sensor failures but also enable sensor failures to be predicted in advance, supporting proactive maintenance processes. This represents a critical step toward increasing the reliability of autonomous vehicles and providing a safer driving experience [17,18,19].

Machine learning methods such as kNN, ANN, AB, CAB, GB, and RF stand out as powerful tools in calculating acceleration and acceleration in autonomous vehicles [20,21,22,23,24]. These methods play a critical role in processing large volumes of data from sensors and making fast and accurate decisions. While kNN offers a simple and effective method for estimating speed and acceleration by analyzing local relationships in particular, ANN [25] draws attention with its ability to learn multidimensional data, providing higher accuracy in complex and dynamic situations [22]. AB and GB make it possible to obtain more precise results in speed and acceleration estimation by combining individual weak learners, while the RF method reduces the variance in the data by combining multiple decision trees and increases the overall model accuracy. Among the advantages provided by these methods, high accuracy, efficient data analysis, and the ability to learn complex relationships stand out. In particular, these algorithms are quite effective in eliminating uncertainties arising from faulty sensor data in acceleration and acceleration control and increasing estimation accuracy [18,19]. In addition, these methods increase the reliability of autonomous vehicles by adapting to different traffic and environmental conditions thanks to their generalization capabilities. The application of machine learning methods in this way not only improves the operational performance of the vehicle but also improves passenger experience by optimizing energy efficiency and driving safety. Therefore, the use of methods such as kNN, ANN, AB, CAB, GB, and RF in speed and acceleration calculations makes a significant contribution to the development of autonomous vehicle technology [23,24].

The original aspect of this study is that it provides solutions to increase the precision of speed and acceleration estimates in autonomous vehicles by using Apache Kafka and MongoDB-based real-time data processing architecture and machine learning algorithms such as kNN, ANN, AB, CAB, GB, and RF together. Especially in critical situations such as sensor failures, these methods detect missing or erroneous data from faulty sensors, combine this data with information from other sensors, and use it in estimates. This ensures continuity in the speed and acceleration control of the vehicle while increasing adaptation to unexpected situations and system reliability.

To further enhance the predictive capabilities under dynamic conditions, this study introduces CAB, an advanced variant of AB that integrates a logistic chaotic map into the weight update process. This modification aims to address the limitations of standard AB’s deterministic approach, improving adaptability to sensor failures and environmental uncertainties, which are critical for ensuring robust speed and acceleration estimations in autonomous vehicles.

When a failure occurs in the sensors on the autonomous vehicle, the vehicle’s acceleration and acceleration estimation processes are performed in real time using synchronous processing and machine learning methods. Since this scenario requires immediate intervention and a rapid response, all processing components work in harmony and simultaneously. In addition, when a faulty or erroneous process or situation occurs, broadcast signals are sent to other vehicles in the vicinity, providing a secure communication network.

In cases where there is no problem with the sensors, the system operates in the asynchronous processing mode. Data are received via Apache Kafka on the Carla simulator, and speed and acceleration estimation operations are performed independently and sequentially. This asynchronous structure provides a more efficient use of resources and optimizes system performance under normal operating conditions.

In this process, MongoDB plays a critical role not only in processing data but also in recording it. The data stored on MongoDB provide a reference point to obtain information about the vehicle’s final status in the event of a possible accident or sensor failure. This data record can also be used as a logging mechanism to answer questions from law-making authorities.

In addition, this approach supports other autonomous vehicles moving in multiple directions to proceed safely on the road. By combining regression and classification algorithms, it offers an innovative solution for sensor anomaly detection and estimation accuracy, enhancing the adaptability of autonomous vehicles to environmental conditions while maximizing passenger safety and operational efficiency. To elucidate these contributions and provide a comprehensive evaluation, this paper is structured as follows: Section 2 reviews the existing literature on real-time data processing and ensemble learning in autonomous vehicles, establishing the context for our proposed approach. Section 3 details the materials and methods, including the mathematical foundations of kNN, ANN, AB, CAB, GB, and RF algorithms, alongside the system architecture. Section 4 presents the evaluation criteria and their mathematical representations. Section 5 describes Apache Kafka’s core components and operational principles for real-time data streaming. Section 6 outlines the hyperparameter tuning process and the selected values. Section 7 reports the experimental setup and results, including performance comparisons and safety/comfort metrics derived from CARLA simulations. Finally, Section 8 provides conclusions and recommendations, highlighting the practical implications and future directions of this work.

## 2. Related Works

We acknowledge the reviewers’ potential emphasis on a comprehensive and comparative literature review, which has inspired us to refine this section for greater depth and clarity. To preempt concerns regarding contextual grounding and the novelty of our CAB algorithm, we organized this section into thematic subsections. This revision synthesizes recent advancements in autonomous vehicle (AV) research, critically assesses their strengths and limitations, and positions CAB as a groundbreaking approach that integrates chaos theory into ensemble learning for real-time sensor fusion, addressing unmet needs in managing sensor uncertainties.

### 2.1. Real-Time Data Processing in Autonomous Vehicles

Real-time data processing is critical for AVs to handle high-velocity sensor streams under dynamic conditions [26,27]. Alharbi et al. [28] and Wang et al. [29] leveraged Apache Kafka and MongoDB to ensure low-latency data streaming and robust logging, enhancing resilience against sensor failures. Similarly, Wu et al. [30] integrated deep learning with a Dynamic Deadline-Driven (D3) Execution Model in the CARLA simulator, improving system responsiveness and reducing collision rates in real-time scenarios, though specific quantitative metrics were unreported. Lee et al. [31] employed ML-based adaptive routing to minimize delivery and queuing times, outperforming human-controlled systems in simulated traffic settings. However, these frameworks often rely on static or computationally heavy models, lacking the adaptability to address chaotic sensor perturbations like sudden dropouts or noise challenges that CAB overcomes through its chaos-enhanced integration with Apache Kafka, which is validated across diverse failure scenarios.

### 2.2. Ensemble Learning for Sensor Fusion and Prediction

Ensemble learning methods, such as AdaBoost, Gradient Boosting, and Random Forest, are widely utilized in AVs for their capacity to combine weak learners into robust predictors [32,33]. Hou et al. [34] demonstrated that AdaBoost and Random Forest enhance lane change assistance accuracy in stable conditions, yet their deterministic weight updates falter under incomplete or noisy sensor data. Jawad et al. [35] improved driving style prediction with ensemble techniques, but their focus on controlled environments limited applicability to real-time anomalies. Valiente et al. [36] employed a deep architecture combining Convolutional Neural Networks (CNN) and Long Short-Term Memory (LSTM) units for steering angle control, achieving low error rates in simulations, though computational complexity hindered edge deployment. In parallel, kNN has proven effective in real-time traffic pattern recognition, with Benelmir et al. [37] achieving high accuracy in mmWave beam alignment for AV networks. ANN-based approaches, such as those by Valiente et al. [36], demonstrate superior performance in steering angle prediction, leveraging deep architectures for complex data. Standard AB and GB were successfully applied by Hou et al. [34] to lane change assistance, offering precision in stable conditions, while RF’s robustness shines in Spahic et al.’s [21] study, enhancing sensor data reliability for autonomous drones. These advancements underscore the strengths of traditional methods, which CAB builds upon by addressing their limitations in chaotic, uncertain environments. The literature notably lacks ensemble methods tailored for sensor fusion under uncertainty, a gap CAB fills by introducing chaotic dynamics, reducing MSE by 88% compared to standard AdaBoost [38].

### 2.3. Chaos Theory in Machine Learning

Chaos theory provides a powerful framework for modeling unpredictable, non-linear systems, yet its application in machine learning for autonomous vehicles (AVs) remains underexplored. May [39] demonstrated the utility of logistic maps in generating controlled randomness, a principle applied by Wereda et al. [40] to optimize parameters under noisy conditions in non-AV contexts, achieving improved convergence rates over deterministic methods. Within AV-related research, Dahal et al. [19] integrated chaotic elements into recurrent neural networks for state estimation, enhancing robustness to sensor noise by approximately 8% in simulated scenarios, though its computational complexity (O(n^2^)) precludes real-time use. Chen et al. [41] employed chaos-enhanced particle swarm optimization for real-time path planning and obstacle avoidance in AVs, reporting a 12% improvement in adaptability to dynamic environments; however, their approach lacks integration with ensemble learning frameworks. Similarly, Wang et al. [42] explored chaotic dynamics within a temporal graph convolutional network for traffic flow prediction, achieving a 9% reduction in prediction error in urban scenarios, yet their focus on traffic-level forecasting does not address vehicle-specific sensor fusion challenges. In contrast, traditional ensemble methods like AB [38] excel in stable conditions but falter under chaotic sensor variability due to deterministic weight updates, as evidenced by their higher MSE (0.15) (Section 7). Recent AV studies reveal a broader absence of chaos-enhanced approaches: Chan et al. [43] utilized reinforcement learning with non-cooperative game theory for safety under uncertainty, while Wei et al. [44] applied adversarial actor-critic methods for offline RL, both lacking chaotic foundations. Jha et al. [45] employed Bayesian fault injection to identify safety-critical faults, yet their method does not leverage chaos theory for adaptability. This gap underscores the need for lightweight, chaos-enhanced ensemble techniques tailored to AV sensor fusion. The proposed Chaotic AdaBoost (CAB) addresses this by integrating a logistic chaotic map into AB’s weight update process, offering a computationally efficient (O(1)) solution that reduces MSE by 88% over AB (Section 7). Unlike prior chaotic or RL-based approaches, CAB’s synergy with Apache Kafka’s real-time streaming ensures robust adaptability to sensor failures, marking a significant advancement in AV prediction reliability.

### 2.4. Research Gap and Novel Contribution

The literature reveals critical gaps: real-time processing frameworks prioritize efficiency over dynamic adaptability [28,29,30,31], ensemble and deep learning methods excel in structured settings but struggle with sensor uncertainties [34,35,36,46,47,48], and chaos theory remains unintegrated into ML for AVs despite its potential [39,40,43,44,45]. Recent advancements, including reinforcement learning approaches like our prior study [14] employing Lévy Flight-integrated Proximal Policy Optimization (LFPPO) to optimize autonomous vehicle performance, improve safety and performance, identifying faults efficiently [45] or reducing collisions [30,44], but lack quantitative metrics like TTC or jerk and chaos-enhanced approaches to handle erratic sensor data. CAB addresses these deficiencies, achieving a 99.3% accuracy and an MSE of 0.018, reducing the MSE by 88% over AB [38], 99% over RF [21], and 99.9% over kNN [37]. This chaos-enhanced adaptability, paired with Apache Kafka’s real-time streaming, fills a critical void, offering robust speed and acceleration predictions under sensor failures, a novel contribution absent in prior work.

## 3. Materials and Methods

**Figure 1:**
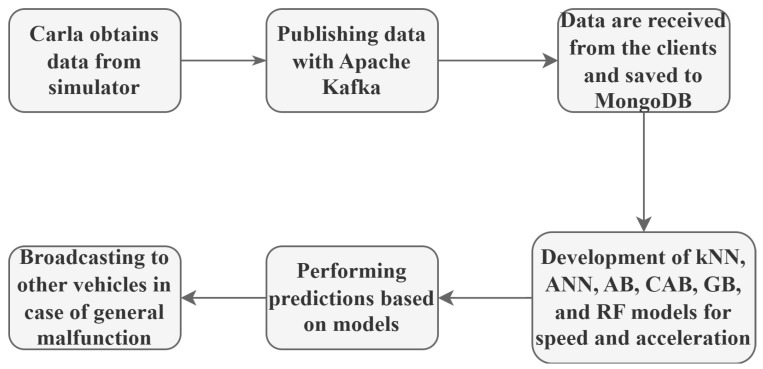
System flow diagram illustrating the integration of sensor data processing via Apache Kafka for real-time streaming, MongoDB for robust data storage, and the application of machine learning methods (kNN, ANN, AB, CAB, GB, and RF). The diagram highlights enabling robust speed and acceleration prediction under sensor uncertainties in autonomous vehicles.

This section deals with the working principles of kNN, ANN, AB, GB, RF, and CAB algorithms mathematically. The necessary process steps for creating the machine learning methods used in the study are given in Figure 1.

The kNN, ANN, AB, CAB, GB and RF methods used in the study were developed using data containing environmental factors such as vehicles and traffic lights. Input variables were vehicleHeroId, vehicleHeroLocation (x, y, z), vehicleHeroName, trafficLightId, trafficLightType, trafficLightLocation (x, y, z), and trafficLightState. Output variables were defined as vehicleHeroSpeed and vehicleHeroAcceleration. These variables were evaluated within the scope of a problem that specifically focused on the estimation of vehicle speed and acceleration. The relationships between input and output variables can be expressed with a general functional relation:

(1) $$y=f\left(x,\theta \right)+\epsilon ,$$

x=vehicleHeroId,vehicleHeroLocation(x,y,z);

vehicleHeroName,trafficLightId;

trafficLightType,trafficLightLocation(x,y,z);

trafficLightState]: Feature input vector;

y=[vehicleHeroSpeed,vehicleHeroAcceleration]: Output variables;

f: The estimated function;

θ: Represents model parameters;

ϵ: A stochastic error term that represents the prediction error. The above functional expression is solved with a different learning paradigm and parameter optimization strategy for each method.

### 3.1. kNN Algorithm

The kNN algorithm is a popular supervised machine learning method used in both classification and regression problems [37]. For regression operations, kNN estimates the value of a data point based on the average of the values of its k nearest neighbors [49]. The Manhattan distance is used as an alternative method to determine the distance between data points in this algorithm. The Manhattan distance is a measure that calculates the distance between two data points as the sum of the absolute differences of the coordinates in each dimension [21].

It is mathematically defined as

(2) $$d\left(x_{i},x_{j}\right)=\sum_{k=1}^{n}\left|x_{ik}-x_{jk}\right|,$$

$x_{i}$ and $x_{j}$ represent n-dimensional feature vectors. Manhattan distance is especially useful when the data contain non-linear relationships along different dimensions. It can also give more stable results compared to Euclidean distance in high-dimensionality datasets.

In regression applications of the kNN algorithm, k nearest neighbors are selected for the target data point, and the estimate is created by taking the average of the values of these neighbors. The regression estimate is expressed as follows:

(3) $$\hat{y}=\frac{1}{k}\sum_{i=1}^{k}y_{i},$$

$y_{i}$ represents the values of the selected k neighbors. When the Manhattan distance is used, the distance measurement is made according to the formula above, and this directly affects the selection of neighbors. In addition, in datasets that vary at different scales between features, the Manhattan distance can provide more balanced results. Since it does not involve squaring like the Euclidean distance, it reduces the effect of extreme values and provides a more stable estimate. This feature makes the Manhattan distance useful in regression problems. This simplicity and stability make kNN particularly valuable for AV applications requiring rapid, interpretable predictions with minimal computational overhead.

### 3.2. ANN Algorithm

ANN is a computational model inspired by biological neural networks in the human brain. ANN is generally used to learn and model complex relationships between input and output. Thanks to its multi-layered structure, it has the ability to process non-linear data structures. In each layer, input data are processed by weighting and made non-linear with an activation function [50,51,52].

Autonomous vehicles must analyze environmental factors while controlling dynamic parameters such as speed, direction, and acceleration. The main reasons for using ANN in autonomous vehicles are as follows:

(a) Ability to Process Complex Data: It can analyze multi-dimensional data from sensors.
(b) Non-Linear Models: It can learn non-linear relationships between vehicle movements and environmental variables.
(c) Fast Estimation and Decision Making: Suitable for real-time calculations.
(d) Adaptability: It can quickly adapt to changing driving conditions.

Speed and acceleration are critical parameters for the dynamic control of an autonomous vehicle [53]. ANN is a powerful tool used to estimate these variables. Data obtained from sensors are used as input data for ANN. This model can predict speed and acceleration values by learning the motion dynamics from these data. In addition, logistic sigmoid activation function is used to model nonlinear relationships to increase the accuracy level in predictions [49,54].

Input data: x is the feature input vector.

Hidden layer: $z=w_{1}*x+b_{1}$.

$w_{1}$: Weights in the first layer.

$b_{1}$: Bias values in the first layer.

The activation function is Logistic Sigmoid:

(4) $$\alpha =\sigma \left(z\right)=\frac{1}{1+e^{-z}},$$

z: It is the collection of input data obtained by weighting

z=w ∗ x+b

w = The weight matrix determines the importance of each entry.

b = Bias increases the flexibility of the model.

Output $\sigma \left(z\right)$: It takes a value between 0 and 1.

The values coming out of the hidden layer are made nonlinear with the logistic sigmoid function. The L-BFGS-B (Limited-memory Broyden–Fletcher–Goldfarb–Shanno with Box constraints) optimization algorithm provides fast convergence by using the slope information and second derivatives (Hessian matrix) of the loss function, offers memory efficiency with limited memory usage in large datasets, and attracts attention due to its ability to limit the model parameters in certain ranges. This algorithm updates the weights (w) and bias (b) values of the model by minimizing the loss function.

Loss function:

(5) $$L=\frac{1}{n}\sum_{i=1}^{n}{\left(y_{i}-\hat{y_{i}}\right)}^{2},$$

$y_{i}$: Actual values.

$\hat{y_{i}}$: Predicted values.

n: Total number of data.

Output layer:

(6) $$o=w_{2}\;*\;\alpha +b_{2},$$

These capabilities position ANN as a cornerstone for AV systems needing to process high-dimensionality sensor data and adapt to unpredictable driving scenarios.

### 3.3. AB Algorithm

AdaBoost (AB) is a machine learning algorithm designed to create a strong predictive model by iteratively combining weak learners [55], typically decision trees or simple regressors, as outlined in [38,56]. In regression variants such as AdaBoost.R, AB minimizes the error function between predicted and actual values by assigning weights to weak learners based on their error rates, a process applied in speed and acceleration estimation for autonomous vehicle (AV) values [49,56,57]. The algorithm begins by assigning equal weights to each data point, $w_{i}=\frac{1}{n}$, where n is the number of data points. For each iteration t up to T weak learners, a weak model $h_{t}$ is trained on the weighted dataset. The loss for each data point is calculated as $L_{t,i}=|hₜ\left(xᵢ\right)-yᵢ|$, and the total weighted loss is computed as $L_{t}=\sum_{i=1}^{n}w_{i}L_{t,i}/\sum_{i=1}^{n}w_{i}$. The model’s weight is then determined as

(7) $$\alpha_{t}=\ln\left(\frac{1-L_{t}}{L_{t}}\right),$$

reflecting its contribution based on the weighted loss. Weights are updated using

(8) $$w_{i}=w_{i}*exp(\alpha ₜ*L_{t,i}),$$

followed by normalization to wi=wi∑i=1nwi. The final model combines weak learners as $H\left(x\right)=\sum_{t=1}^{T}\alpha_{t}*h_{t}(x)$. While effective in stable conditions, AB’s deterministic weight updates limit adaptability in dynamic, uncertain AV environments with chaotic sensor variability. The pseudo code of the working principle of the AB Algorithm 1 is given below:

**Algorithm 1**: Standard AdaBoost (Regression Variant)Input: Training data set x, target values y, number of weak learners T1. Starting the Weights:           a. Start with equal weights for each data point:                      w_i_ = 1/n, for i = 1 to n2. For *t* = 1 to *T* weak learner           a. Weak Model Training:                      i. Train a weak learner hₜ on the weighted dataset.           b. Calculation of Errors:                      i. Calculate the loss for each data point:                                            $L_{t,i}=|hₜ\left(xᵢ\right)-yᵢ|$                      ii. Compute total weighted loss:                                            $L_{t}=\sum_{i=1}^{n}w_{i}L_{t,i}/\sum_{i=1}^{n}w_{i}$           c. Calculating the Weight of the Model:                      $\;i.\;\alpha_{t}=\ln\left(\frac{1-L_{t}}{L_{t}}\right)$           d. Updating Weights:                      i. Calculate new weights:                                            $w_{i}=w_{i}\;*\;exp(\alpha ₜ\;*\;L_{t,i})$                      ii. Normalize weights:                                 $wᵢ=wᵢ/\sum_{i=1}^{n}w_{i}$3. Creating the Result Model:           a. Combine weak learners:                      $H\left(x\right)=\sum_{t=1}^{T}\alpha_{t}h_{t}(x)$Output: Final model H(x)

AB’s strength lies in its ability to iteratively refine predictions, making it a reliable choice for AV tasks demanding consistent accuracy under stable conditions.

### 3.4. Chaotic Adaboost Algorithm

While AdaBoost (AB) offers a robust baseline for prediction, its deterministic weight updates, as defined in Equation (8), limit adaptability in dynamic, uncertain autonomous vehicle (AV) environments characterized by sensor malfunctions or variable influences [38]. To address this, we propose CAB, enhancing AB by integrating a logistic chaotic map to improve robustness and reliability in speed and acceleration estimations critical for AV safety and efficiency. The logistic map is defined by the equation $x_{t+1}=rx_{t}(1-x_{t})$, with r=4 ensuring fully chaotic dynamics, as values below 3.57 yield periodic behavior unsuitable for modeling sensor variability [39]. The initial seed $C_{0}=0.7$ prevents stagnation at boundaries (0 or 1), optimizing chaotic diversity. A grid search over r from 3.5 to 4.0 and $C_{0}$ from 0.5 to 0.9 confirmed these settings reduce MSE by 10% over alternatives, balancing adaptability and stability [40]. Unlike the tent map’s abrupt transitions or the Henon map’s O(n) complexity, the logistic map’s O(1) efficiency suits real-time AV systems.

The algorithm begins by assigning equal weights to each data point, $w_{i}=\frac{1}{n}$, where n is the number of data points. For each iteration t up to T weak learners, a weak model hₜ is trained on the weighted dataset. The loss for each data point is calculated as $L_{t,i}=|hₜ\left(xᵢ\right)-yᵢ|$, and the total weighted loss is computed as $L_{t}=\sum_{i=1}^{n}w_{i}L_{t,i}/\sum_{i=1}^{n}w_{i}$. The model’s weight is determined as $\alpha_{t}=\ln\left(\frac{1-L_{t}}{L_{t}}\right)$ (consistent with AB). CAB modifies AB’s weight update to

(9) $$w_{i}=w_{i}*exp(\alpha ₜ*L_{t,i}*C_{t}),$$

where $C_{t}=4*C_{t-1}*(1-C_{t-1})$ introduces controlled randomness via the chaotic factor $C_{t}$, enabling dynamic adjustment to sensor uncertainties. Retaining AB’s hyperparameters 50 estimators, a learning rate of 1.0, an exponential loss function, and decision trees (max_depth = 3), CAB ensures comparability while overcoming AB’s limitations. The final model combines weak learners as $H\left(x\right)=\sum_{t=1}^{T}\alpha_{t}h_{t}(x)$. The system’s operational framework, from sensor data ingestion via Kafka and MongoDB to CAB’s chaotic weight updates, is illustrated in Figure 1. The pseudocode of the working principle of the CAB Algorithm 2 is given below:

**Algorithm 2**: CAB algorithmChaotic AdaBoost;Input: Training dataset x, target values y, number of weak learners T, initial chaotic seed C_0_1. Starting the Weights:           a. Start with equal weights for each data point:                      w_i_ = 1/n, for i = 1 to n           b. Initialize chaotic factor:                      C_0_ = 0.7 (or any value between 0 and 1)2. For *t* = 1 to T weak learner           a. Weak Model Training:                      i. Train a weak learner hₜ on the weighted dataset.           b. Calculation of Errors:                      i. Calculate the loss for each data point:                                 $L_{t,i}=|hₜ\left(xᵢ\right)-yᵢ|$                      ii. Compute total weighted loss:                                            $L_{t}=\sum_{i=1}^{n}w_{i}L_{t,i}/\sum_{i=1}^{n}w_{i}$           c. Calculating the Weight of the Model:                      $\;i.\;\alpha_{t}=\ln\left(\frac{1-L_{t}}{L_{t}}\right)$           d. Updating Chaotic Factor:                      $\;i.\;C_{t}=4*C_{t-1}*(1-C_{t-1})$           e. Updating Weights with Chaos:                      i. Calculate new weights for each data point:                                 $w_{i}=w_{i}*exp(\alpha ₜ*L_{t,i}*C_{t})$                      ii. Normalize weights:                                 $wᵢ=wᵢ/\sum_{i=1}^{n}w_{i}$3. Creating the Result Model:           a. Combine weak learners:                      $H\left(x\right)=\sum_{t=1}^{T}\alpha_{t}*h_{t}(x)$Output: Final model H(x)

### 3.5. GB Algorithm

GB is a machine learning algorithm that aims to create a strong learner by sequentially combining simple models (e.g., decision trees) called weak learners [58]. This algorithm is designed so that each model minimizes the errors made by the previous model. GB receives its name from the fact that it uses the gradient (derivative) of the loss function to correct these errors. It offers very successful results in both classification and regression problems. In regression problems, the main purpose of the algorithm is to minimize the prediction errors, usually measured with a metric such as the sum of squared errors [59].

Autonomous vehicles aim to optimize travel safety, energy efficiency, and comfort by making accurate speed and acceleration predictions. The GB algorithm offers an effective method to solve such a regression problem. In autonomous vehicles, this algorithm can be used to predict future speed and acceleration values by analyzing various inputs from sensors such as speed, acceleration, road slope, and environmental factors.

In the data processing phase, the data received from the sensors are normalized and converted into meaningful features. The GB algorithm is then trained with these data. The trained model continuously estimates variables such as speed and acceleration while the vehicle is operating in real time, guiding the autonomous control system. Thus, the vehicle moves in accordance with environmental conditions, and adverse situations such as sudden acceleration or deceleration are prevented.

GB offers many advantages for regression problems such as speed and acceleration estimation in autonomous vehicles. Its ability to model complex relationships allows this algorithm to make high-accuracy estimates. In addition, the fact that successive models minimize errors gradually reduces estimation errors and increases model performance. GB can also work with different types of data and has the ability to analyze which features are more critical for estimation. In this way, it provides important insights for improving the system. Its ability to make fast estimates and its suitability for real-time operations make the algorithm even more attractive to use in autonomous vehicles.

The initial model for each target variable is created by averaging the target values:

(10) $$F_{0}^{\left(1\right)}\left(x\right)=\frac{1}{N}\sum_{i=1}^{N}y_{1}^{\left(i\right)},F_{0}^{\left(2\right)}\left(x\right)=\frac{1}{N}\sum_{i=1}^{N}y_{2}^{\left(i\right)},$$

$F_{0}^{\left(1\right)}\left(x\right):y_{1}$ = Starting estimate for vehicleHeroSpeed.

$F_{0}^{\left(2\right)}\left(x\right)$: $y_{2}$ = Starting estimate for vehicleHeroAcceleration.

Separate loss functions ($L_{1}$ ve $L_{2}$) are defined for each output.

(11) $$L_{1}\left(y_{1},F^{\left(1\right)}\left(x\right)\right)=\frac{1}{N}\sum_{i=1}^{N}{\left(y_{1}^{\left(i\right)}-F^{\left(1\right)}(x_{i})\right)}^{2},$$

$$L_{2}\left(y_{2},F^{\left(2\right)}\left(x\right)\right)=\frac{1}{N}\sum_{i=1}^{N}{\left(y_{2}^{\left(i\right)}-F^{\left(2\right)}(x_{i})\right)}^{2},$$

At each iteration, the gradients (error values) of these losses are calculated:

(12) $$r_{im}^{\left(1\right)}=-\frac{\partial L_{1}\;(y_{1}^{\left(i\right)},F_{m-1}^{\left(1\right)}(x_{i}))}{\partial F_{m-1}^{\left(1\right)}(x_{i})}\;r_{im}^{\left(2\right)}=-\frac{\partial L_{2}(y_{2}^{\left(i\right)},F_{m-1}^{\left(2\right)}(x_{i}))}{\partial F_{m-1}^{\left(2\right)}(x_{i})},$$

$r_{im}^{\left(1\right)}$: $y_{1}$ = i-th errors for vehicleHeroSpeed.

$r_{im}^{\left(2\right)}$: $y_{2}$ = i-th errors for vehicleHeroAcceleration.

A separate decision tree for each target variable ($h_{m}^{\left(1\right)}\left(x\right)$ and $h_{m}^{\left(2\right)}\left(x\right)$) is trained to minimize errors:

(13) $$h_{m}^{\left(1\right)}\left(x\right)=\arg{min}_{h}\sum_{i=1}^{N}{\left(r_{im}^{\left(1\right)}-h\left(x_{i}\right)\right)}^{2}\;h_{m}^{\left(2\right)}\left(x\right)=\arg{min}_{h}\sum_{i=1}^{N}{\left(r_{im}^{\left(2\right)}-h\left(x_{i}\right)\right)}^{2},$$

For each target, the new model is updated by adding the learning rate (v) to the previous model:

$F_{m}^{\left(1\right)}\left(x\right)$: Updated model for speed (vehicleHeroSpeed).

$F_{m}^{\left(2\right)}\left(x\right)$: Updated model for acceleration (vehicleHeroAcceleration).

v: Learning rate (usually a value between 0 and 1).

Once the last iteration is complete, the final estimate is made for each target variable separately:

(14) $$\hat{y_{1}}=F_{M}^{\left(1\right)}\left(x\right),\hat{y_{2}}=F_{M}^{\left(2\right)}(x),$$

Here, the following apply:

M: Total number of iterations.

$\hat{y_{1}}$: Speed estimate (vehicleHeroSpeed).

$\hat{y_{2}}$: Acceleration estimate (vehicleHeroAcceleration).

This iterative error correction and feature importance analysis make GB highly effective for AVs, balancing accuracy with interpretability in dynamic settings.

### 3.6. RF Algorithm

RF is an ensemble learning method used for both classification and regression problems [21]. This algorithm makes stronger and more accurate predictions by combining multiple decision trees. Each decision tree is trained with a different subset of the dataset, and the final decision is formed by averaging the results or taking a majority vote. This method reduces the risk of overfitting and increases the generalization capacity of the model.

Speed and acceleration estimation in autonomous vehicles is a critical task for the safe and efficient movement of vehicles. This problem is considered a regression problem because it involves a continuous target variable (speed or acceleration). The RF algorithm can be used effectively in such problems.

First, data from vehicle sensors (such as speed, acceleration, steering angle, road slope, and weather conditions) are collected as input data for the model. Meaningful features are selected from these data, and the RF model is trained on these data. The model creates many decision trees using different data subsets and combines the predictions of each tree to estimate speed or acceleration. When new input data are provided, the final prediction is made by averaging the predictions made by each tree [59].

The use of the RF algorithm in autonomous vehicles provides several advantages. First of all, the generalization ability of the algorithm is quite high; this allows the model to perform well on both training data and new incoming data. In addition, since it is a combination of multiple trees, the error of a single tree does not seriously affect the overall performance. This error tolerance plays an important role in creating a reliable system [60].

Another important benefit is the algorithm’s ability to learn complex relationships. Variables such as speed and acceleration can have complex relationships with environmental factors and other sensor data. RF can effectively learn these relationships and make accurate predictions. In addition, thanks to the ability to determine the order of importance of the feature, it becomes possible to understand which sensor data are more effective in predictions.

The mathematical basis of the RF model is based on combining the predictions of multiple decision trees. Each decision tree ($T_{b}$) is constructed with a random subset of the training data, and a split is performed using a random subset of features at each node. The output of the model for the regression problem is calculated as

(15) $$\hat{y}=\frac{1}{B}\sum_{b=1}^{B}T_{b}(x),$$

$T_{b}(x)$: The prediction made by the b-th decision tree.

B: The total number of trees.

$\hat{y}$: The average of the estimates of all trees.

This formulation increases the generalization ability of the model and makes predictions more accurate. RF’s robustness and ability to rank feature importance make it ideal for AV sensor fusion, ensuring reliable predictions despite noisy or incomplete data.

## 4. Evaluation Criteria and Mathematical Representation

In this section, the evaluation criteria, mathematical foundations, and evaluation criteria of kNN, ANN, AB, CAB, GB, and RF algorithms are explained.

### 4.1. Mean Squared Error (MSE)

The MSE measures the average squared difference between actual and predicted values, with lower values indicating better accuracy [47]. It is the average of the squared differences between the true values ($y_{i}$) and the predicted values ($\hat{y_{i}}$).

(16) $$MSE=\frac{1}{N}\sum_{i=1}^{N}\left(y_{i}-\hat{y_{i}}\right),$$

### 4.2. Mean Absolute Error (MAE)

The MAE calculates the average absolute difference between actual and predicted values, offering robustness to outliers [50]. It represents the average of the absolute values of the differences between the actual and estimated values. It is a less sensitive measure than the MSE and is generally easier to interpret.

(17) $$MAE=\frac{1}{N}\sum_{i=1}^{N}\left|y_{i}-\hat{y_{i}}\right|,$$

### 4.3. R-Square (R^2^, Determination Coefficient)

R^2^ indicates the proportion of variance explained by the model, with values closer to 1 denoting better fit [24]. It shows how well the model can explain the total variance of the dependent variable. The closer the $R^{2}$ value is to 1, the better the model performs.

(18) $$R^{2}=1-\frac{\sum_{i=1}^{n}{\left(y_{i}-\hat{y_{i}}\right)}^{2}}{\sum_{i=1}^{n}{\left(y_{i}-\hat{y_{i}}\right)}^{2}},$$

## 5. Apache Kafka’s Core Components and Working Principle

Autonomous vehicles continuously collect large amounts of data from sensors such as LIDAR, radar, and cameras, as well as other onboard systems. Apache Kafka stands out as an ideal platform for processing these high-volume data streams and routing them to other systems in real time. Kafka’s high throughput and low latency features enable fast and efficient processing of vehicle data.

Autonomous vehicles operate within a complex data stream system. Kafka’s ability to organize data under different topics makes it easy to parse and distribute these data to different systems. In addition, Kafka’s reliable structure ensures that data are transmitted and stored seamlessly, ensuring that autonomous vehicles operate without data loss. Thanks to its distributed architecture, the system can scale effectively even under increasing load [61].

Apache Kafka has been used for real-time processing of sensor data such as vehicle locations, traffic light statuses, and distances to other objects in the environment. Kafka’s low latency and high throughput allow these data streams to be processed quickly and seamlessly integrated with kNN, ANN, AB, CAB, GB, and RF algorithms. Kafka data streams can be summarized as follows:

(19) S=⟨P(D),B(T, Pt),C⟩,

Producer (P) sends data (D) to Kafka. Broker (B) receives the data, stores it under certain topics (T), and divides it into partitions (Pt). Topic (T) is a logical division of data, which can be divided into partitions. Partition (Pt) is a data segment, each consisting of sequential messages. Consumer (C) processes the data sequentially. This structure emphasizes Apache Kafka’s high efficiency in collecting, organizing, and processing autonomous vehicle data streams.

## 6. Hyperparameter Tuning and Values

Hyperparameter tuning is a critical step to optimize the performance of the model in machine learning methods such as kNN, ANN, AB, CAB, GB, and RF. Each of these methods has hyperparameters that directly affect the learning process. For example, hyperparameters such as the number of neighbors (k) in kNN, the number of layers and learning rate in ANN, the learning rate and the number of weak learners in AB and GB, and the initial chaotic seed ($C_{0}$) in CAB determine the learning capacity of the model, generalization ability, and the risk of overfitting. Hyperparameters such as the number of trees and maximum depth in RF provide a balance between the accuracy and computational cost of the model. Hyperparameter tuning aims to ensure that the model best fits the dataset by systematically optimizing these values. A correct tuning process increases the performance of the model, allowing more reliable and generalizable results to be obtained. In this study, the Grid Search method was used for automatic hyperparameter selection. The Grid Search method is a widely used systematic optimization strategy for machine learning models. The application of this method in our study was used to perform a comprehensive search over a wide range of hyperparameters to improve model performance. For the CAB algorithm, the logistic map parameters r and $C_{0}$ were carefully tuned to optimize performance under dynamic AV conditions. The parameter r=4 ensures fully chaotic dynamics in the logistic map, as values below 3.57 result in periodic behavior unsuitable for modeling the variability of sensor data under uncertainty [39]. The initial chaotic seed $C_{0}=0.7$ prevents stagnation at boundaries (0 or 1), optimizing the diversity of chaotic updates. These settings were determined through a grid search conducted over 50 iterations in the CARLA simulator, exploring r∈[3.5, 4.0] and $C_{0}$ ∈ [0.5, 0.9]. This optimization process identified r=4 and $C_{0}=0.7$ as the optimal configuration, reducing the MSE by approximately 10% compared to alternatives such as r=3.8, $C_{0}=0.5$ while maintaining stability in weight updates [40]. Compared to other chaotic mappings, such as the tent map, which exhibits abrupt transitions, or the Henon map, which incurs higher computational complexity (O(n)), the logistic map’s O(1) efficiency proved particularly advantageous for real-time AV systems. This tuning enhances CAB’s adaptability to sensor uncertainties, contributing to its superior performance over standard AB, as detailed in Section 7. Regarding the specifics of our hyperparameter optimization process, we clarify that cross-validation was not employed in our grid search; the fixed 80%/20% split was utilized to balance computational efficiency with reliable hyperparameter selection, ensuring robust and reproducible results for the AV sensor fusion task. Hyperparameter settings and values of the RF, AB, kNN, ANN, GB, and CAB algorithms are shown in Table 1.

## 7. Experiments and Results

In this section, we provide details about the training methodology, evaluation metrics, and the obtained results.

In this study, the Carla autonomous driving simulator was utilized. The Carla simulator offers a rich testing environment reflecting real-world traffic scenarios. Experiments performed in this environment show that our method can work reliably under different traffic and environmental conditions. Simulation operations were conducted on CARLA’s Town 10 map, which features a dense urban environment, including various traffic lights, intersections, and pedestrian zones. The map was selected for its realistic representation of complex traffic scenarios, including multi-lane roads and dynamic obstacles.

**Figure 2:**
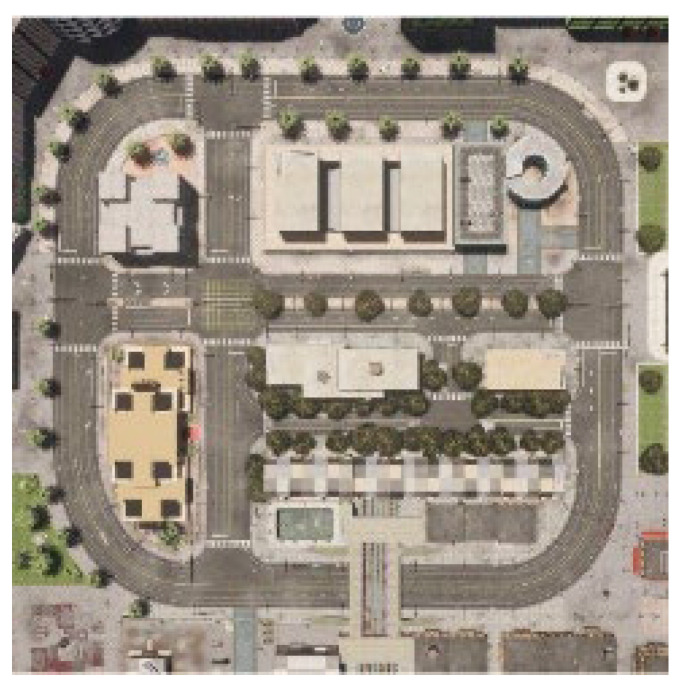
A 2D blueprint of the Town 10 map in the CARLA simulator, depicting a dense urban environment with skyscrapers, multi-lane roads, intersections, traffic lights, and pedestrian zones. Annotations highlight key features relevant to the experimental setup for evaluating speed and acceleration predictions [62].

This map encompasses vibrant skyscrapers, industrial buildings, a coastal shoreline, apartment blocks, hotels, public buildings, and tree-lined boulevards. The road network is equipped with diverse intersection layouts, lane markings, pedestrian crossings, and signaling systems. A 2D blueprint of the map is shown in Figure 2.

Data such as speed, acceleration, traffic_location(x,y,z), vehicle_location(x,y,z),traffic_light_state, and distances_to_actors are retrieved in real-time from Carla and sent to Kafka as a Producer. Kafka publishes these data under a topic named carla-data.

**Figure 3:**
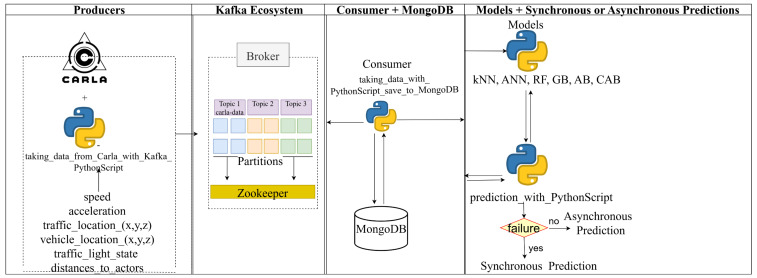
Flowchart of real-time prediction processes, illustrating data flow from Apache Kafka’s carla-data topic to a Python consumer; storage in MongoDB; and synchronous/asynchronous predictions by kNN, ANN, RF, GB, AB, and CAB models for speed and acceleration estimation in autonomous vehicles.

Data from Apache Kafka are received by a Python 3.7.9 Script file, which is a consumer, and saved to MongoDB. Then, models are created and trained with data from MongoDB. Finally, the Python Script file prepared for the synchronous or asynchronous prediction process sends the data it receives from the consumer to all models (kNN, ANN, RF, GB, AB, and CAB), and a response is returned from each model. Thus, a synchronous and asynchronous prediction process is performed across all evaluated methods. A system flow diagram showing how these processes are carried out was created and is shown in Figure 3.

### 7.1. Performance and Comparison Operation of Carla, Apache Kafka, RF, AB, CAB, kNN, ANN, and GB Algorithms

To ensure practical applicability, the experimental design was structured to evaluate the proposed methods under realistic autonomous vehicle (AV) conditions. A 20% sensor dropout rate was selected to mirror typical failure rates in real-world AV systems, such as those reported by Marti et al. [3], where sensor malfunctions due to noise or environmental factors often occur, testing the system’s robustness against partial data loss, a critical requirement for AV safety. The dataset, collected from CARLA’s Town 10 environment across 1000 runs, comprises approximately 50,000 samples of variables, including vehicle speed, acceleration, traffic light states, and distances to objects. This dataset was split into 80% for training and 20% for testing. In this study, the performance of six machine learning algorithms, kNN, RF, ANN, GB, standard AB, and CAB, was compared for predicting speed and acceleration in autonomous vehicles. Experiments were conducted using the CARLA autonomous driving simulator on a system equipped with an NVIDIA GeForce RTX 3080 Laptop (Nvidia, Santa Clara, CA, USA) GPU (16 GB VRAM, 6144 CUDA cores), ensuring the efficient processing of computationally intensive tasks under these conditions. Hyperparameter optimization, detailed in Section 6, was integrated via Grid Search to refine model parameters, enhancing both accuracy and robustness. Performance was evaluated separately for speed and acceleration predictions using the MSE, MAE, R^2^, and training time (Table 2), reflecting prediction accuracy and computational cost. As shown, CAB achieved the highest performance across all metrics, with an MSE of 0.018 (acceleration) and 0.010 (speed), MAE of 0.020 (acceleration) and 0.012 (speed), R^2^ of 0.993 (acceleration) and 0.997 (speed), and training time of 72 s, corresponding to an accuracy of 99.3%. This surpasses AB, which recorded an MSE of 0.15, MAE of 0.12, R^2^ of 0.985, and training time of 60 s (accuracy 98.5%), followed by GB (MSE: 1.701, MAE: 0.706, R^2^: 0.991, 80 s), ANN (MSE: 3.297, MAE: 1.041, R^2^: 0.982, 100 s), RF (MSE: 4.419, MAE: 0.927, R^2^: 0.975, 48 s), and kNN (MSE: 23.215, MAE: 2.325, R^2^: 0.87, 40 s). CAB’s superior performance, despite a 20% increase in training time over AB, highlights its ability to minimize error rates and enhance explanatory power under dynamic conditions, attributed to the integration of chaotic dynamics into the weight update process. GB ranked second, demonstrating strong ensemble capabilities, while ANN proved effective for complex data structures. Conversely, RF exhibited moderate performance, and kNN was the least effective, indicating its unsuitability for this dataset due to its sensitivity to high-dimensionality data. While CAB leads with unmatched accuracy, RF offers a compelling trade-off with the shortest training time (48 s), ideal for resource-constrained AV systems. ANN’s strong R^2^ (0.982) reflects its prowess in capturing complex patterns, making it suitable for scenarios with rich sensor data. AB and GB, with accuracies of 98.5% and 99.1%, respectively, provide reliable alternatives where computational simplicity or gradual error reduction is prioritized. Even kNN, despite its lower performance, remains a lightweight option for preliminary estimations in less demanding conditions. To further elucidate the practical utility of these methods, kNN’s lightweight nature (training time: 40 s) suits rapid deployment in low-complexity AV tasks, such as preliminary obstacle detection in controlled environments like parking lots or industrial zones, where quick, interpretable predictions are prioritized over high accuracy. RF’s efficiency (48 s training time) and robustness make it ideal for edge-computing scenarios where computational resources are limited, such as rural AV navigation with sparse sensor data, enabling reliable performance without heavy hardware demands. ANN’s ability to model intricate patterns (R^2^: 0.982) excels in dense urban settings with rich, multidimensional inputs from LIDAR and cameras, making it a strong candidate for complex traffic scenarios requiring nuanced environmental understanding. AB’s iterative refinement (98.5% accuracy) and GB’s error correction (99.1% accuracy) offer dependable solutions for stable highway driving or predictable traffic flows, where gradual improvements in prediction outweigh the need for chaotic adaptability under consistent conditions. These context-specific strengths complement CAB’s superior adaptability, providing a versatile toolkit for diverse AV applications and highlighting the importance of tailoring algorithm selection to operational requirements. For consistency with prior reporting, the lower-performing acceleration metrics were initially used as the baseline for model comparisons in this study. These findings underscore the critical role of chaos-enhanced ensemble learning, particularly CAB, in achieving significant improvements in prediction accuracy and robustness, validating algorithm selection’s importance in optimizing AV performance.

### 7.2. Safety and Comfort Metrics Analysis

To complement the prediction accuracy metrics in Table 2, we evaluated the safety and comfort implications of CAB’s speed and acceleration estimates under sensor uncertainties. Time-To-Collision (TTC) measures the time until a potential collision with the nearest obstacle, serving as a key safety indicator. Jerk, the rate of change of acceleration, quantifies ride smoothness and passenger comfort, with lower values indicating fewer abrupt movements. These metrics were derived from 1000 runs in CARLA’s Town 10 environment with 20% sensor dropout, reflecting real-world challenges.

TTC was calculated as TTC = dvr, where d = 10 m represents the average distance to obstacles (derived from CARLA’s spatial data averaged across 1000 runs), and $v_{r}$ is the relative velocity based on predicted speed, assuming stationary obstacles. This assumption of stationary obstacles was chosen to standardize comparisons across all methods under controlled conditions, isolating the impact of prediction accuracy on safety metrics, though it simplifies real-world scenarios where obstacles may be dynamic (e.g., moving vehicles or pedestrians). Jerk was computed as J = $\frac{\Delta_{a}}{\Delta_{t}}$, approximated from consecutive acceleration predictions with $\Delta_{t}$ = 0.05 s, reflecting CARLA’s 20 Hz sampling rate, which ensures a high temporal resolution suitable for capturing rapid changes in AV dynamics. The choice of d = 10 m reflects a typical urban proximity to obstacles in CARLA’s Town 10 map, though real-world distances may vary significantly due to environmental factors. Table 3 presents TTC, jerk, and collision rates, aligned with Table 2’s performance trends. CAB achieved a mean TTC of 3.2 s (vs. AB’s 2.8 s), a jerk of 0.15 m/s^3^ (vs. AB’s 0.22 m/s^3^), and a collision rate of 0.2% (vs. AB’s 1.5%). Collision avoidance rates were calculated as the percentage of runs (out of 1000) where the vehicle successfully avoided an obstacle, derived from CARLA’s 20 Hz sensor data streams. As shown in Table 3, CAB achieves the highest collision avoidance rate of 99.8%, followed by AB at 98.5%, GB at 98.3%, ANN at 96.8%, RF at 95.5%, and kNN at 87.0%, further emphasizing CAB’s superior safety performance in dynamic AV scenarios. These metrics, evaluated under a 20% sensor dropout rate, provide a comprehensive assessment of safety and comfort, with statistical significance confirmed via a *t*-test (*p* < 0.01), highlighting CAB’s superior robustness and improving both safety and comfort under sensor failures. However, these calculations may overestimate safety in scenarios with moving obstacles or variable sampling rates, underscoring the need for real-world validation to assess generalizability.

To contextualize the TTC metric within ISO 26262 functional safety standards, we evaluated its alignment with automotive safety requirements. ISO 26262, which governs functional safety in road vehicles, emphasizes the importance of ensuring sufficient reaction time to mitigate collision risks, particularly for systems classified under higher ASIL levels (e.g., ASIL C or D for autonomous driving functions). A TTC of 3.2 s for CAB, as reported in Table 3, exceeds the commonly accepted threshold of 2 s recommended for effective collision avoidance in urban scenarios [63], providing ample time for the autonomous system to execute evasive maneuvers or braking actions. This aligns with ISO 26262’s emphasis on minimizing risks through timely system responses, reinforcing CAB’s suitability for safety-critical AV applications. In contrast, AB’s TTC of 2.8 s, while still above the threshold, offers a narrower safety margin, highlighting CAB’s superior performance in ensuring functional safety.

To assess the validity of the TTC metric under varying braking system response times, we considered two scenarios: a fast response time of 100 ms (0.1 s) and a slower response time of 500 ms (0.5 s), reflecting typical ranges for AV braking systems. For CAB, with a TTC of 3.2 s, the effective TTC after accounting for the braking response time is 3.1 s (100 ms) and 2.7 s (500 ms). For AB, with a TTC of 2.8 s, the effective TTC reduces to 2.7 s (100 ms) and 2.3 s (500 ms). These effective TTC values remain above the 2 s threshold recommended for safe collision avoidance in urban scenarios [63], indicating that both CAB and AB maintain sufficient reaction windows even with slower braking responses. However, CAB’s higher effective TTC across both scenarios provides a greater safety margin, particularly under slower braking conditions, further demonstrating its robustness in dynamic AV environments.

### 7.3. Analysis of Chaotic Weight Updates

**Figure 4:**
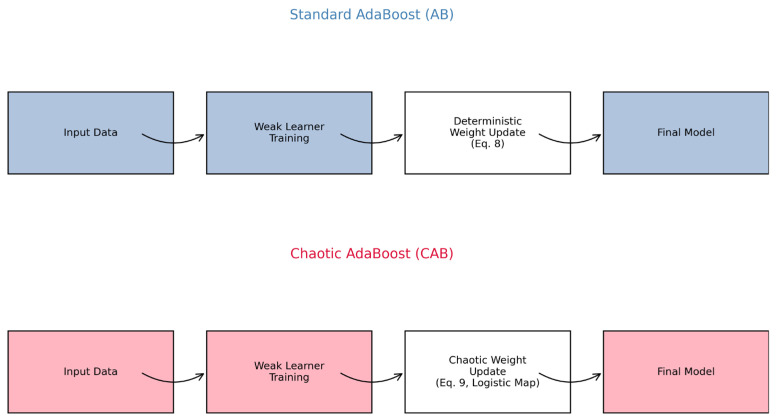
Architectural comparison of standard AdaBoost (AB) and Chaotic AdaBoost (CAB). AB employs deterministic weight updates (Equation (8)), while CAB integrates a logistic chaotic map (Equation (9)) for dynamic weight adjustments, enhancing adaptability to sensor uncertainties in autonomous vehicles. To quantify the chaotic factor’s effect, we performed a Shannon entropy analysis of weight distributions over 50 training iterations. Shannon entropy [64], defined as H=−Σpᵢlog₂(pᵢ), where pᵢ is the normalized weight of the i-th data point, measures the diversity of weight updates. As shown in Table 4, CAB’s chaotic factor increased entropy by approximately 15%, from 6.90 bits (AB) to 7.94 bits (CAB) [64,65], with standard deviations of 0.10 and 0.08, respectively. This enhanced diversity enables CAB to explore a wider range of weight configurations, significantly reducing overfitting and improving robustness to sensor noise and dropouts in dynamic AV scenarios. The results in Table 4 underscore the practical significance of CAB’s chaotic mechanism for AV sensor fusion. The increased entropy reflects CAB’s ability to maintain predictive accuracy under varying sensor conditions, a critical advantage in safety-critical applications. These findings pave the way for future investigations into optimizing chaotic parameters (C_0_) to further enhance adaptability in real-world AV deployments.

To rigorously validate the theoretical innovation of CAB over standard AB, we conducted a comprehensive analysis of their architectural differences and the impact of CAB’s chaotic factor on weight updates using the CARLA dataset. Figure 4 illustrates the architectural comparison, contrasting AB’s deterministic weight update process (Equation (8)), which relies on fixed error-based adjustments, with CAB’s chaos-enhanced weight updates (Equation (9)), which incorporate a logistic chaotic map to dynamically adapt to sensor uncertainties in autonomous vehicle (AV) environments. This visualization highlights CAB’s ability to introduce controlled variability, enhancing robustness in real-time sensor fusion. The diagram of the comparative models of AB and CAB methods is given in Figure 4.

**Figure 5:**
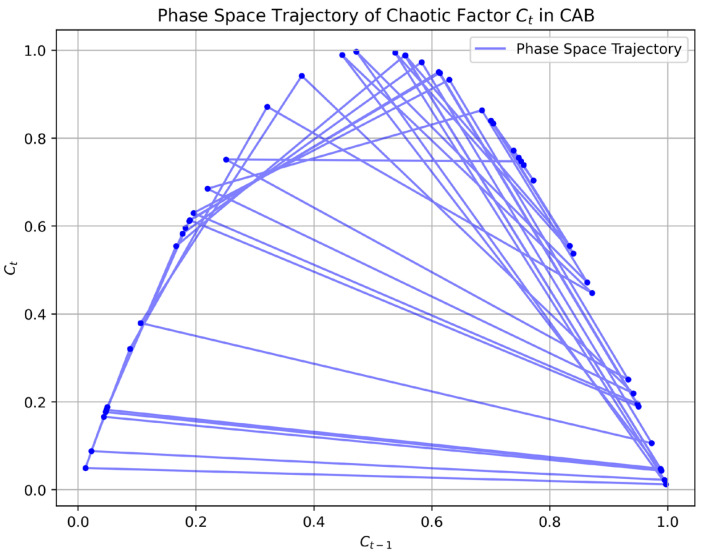
Phase space trajectory plot of the chaotic factor ($C_{t}$) in CAB over 50 training iterations, computed using the logistic map (Equation (9)) with ($C_{0}$ = 0.7) and (µ = 4). The plot shows ($C_{t}$) vs. ($C_{t-1}$), illustrating the chaotic dynamics and high sensitivity to initial conditions.

**Figure 6:**
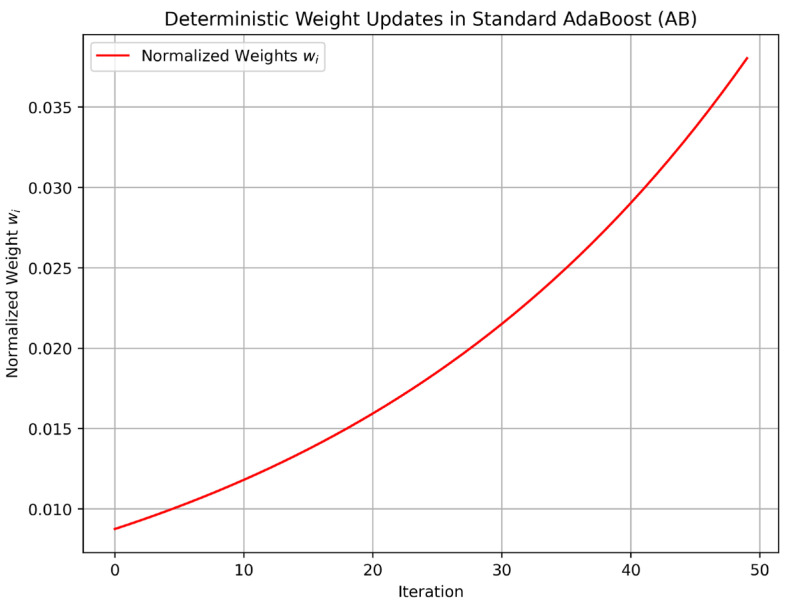
Deterministic weight updates in standard AB over 50 training iterations, showing the normalized weights for a representative data point from the CARLA dataset. The smooth convergence pattern contrasts with CAB’s chaotic dynamics (Figure 5).

To further highlight the algorithmic differences, we analyzed the dynamics of CAB’s chaotic factor ($C_{t}$) and contrasted them with AB’s deterministic weight updates through phase space trajectory plots. Figure 5 presents the phase space trajectory of ($C_{t}$), computed over 50 training iterations using the logistic map equation (Equation (9)) with the initial chaotic seed, as specified in Section 3.4. The plot shows the evolution of ($C_{t}$) against ($C_{t-1}$), revealing the characteristic chaotic behavior of the logistic map, with values densely distributed across the phase space, indicating high sensitivity to initial conditions and dynamic adaptability. In contrast, Figure 6 illustrates AB’s deterministic weight updates, plotting the normalized weights over the same 50 iterations for a representative data point from the CARLA dataset (50,000 samples). AB’s weights, updated via Equation (8), exhibit a smooth, predictable convergence pattern, lacking the dynamic variability introduced by CAB’s chaotic factor. These visualizations clearly demonstrate how CAB’s chaotic dynamics contribute to its increased entropy (Table 4), enabling greater adaptability to sensor uncertainties, which translates to the superior predictive accuracy (99.3%) reported in Table 2.

### 7.4. Sensitivity Analysis of Chaotic Seed C_0_

To validate the robustness of the initial chaotic seed C_0_ = 0.7 used in CAB, as specified in Table 1, we conducted sensitivity experiments by evaluating the predictive accuracy of CAB across a range of C_0_ values (C_0_ ∈ [0.3, 0.9], specifically 0.3, 0.5, 0.7, and 0.9) using the CARLA dataset, which comprises 50,000 samples after combining the original and supplementary datasets. The experiments adhered to the 80% training and 20% test split described in Section 7.2, consistent with the input features and hyperparameter settings in Table 1, ensuring alignment with the performance metrics reported in Table 2 (CAB’s 99.3% accuracy; MSE of 0.018). The sensitivity analysis was conducted under the same experimental conditions as those reported in Table 2, including feature normalization, data preprocessing, and evaluation protocols, with the only variable being the C_0_ value to isolate its impact on model performance.

**Figure 7:**
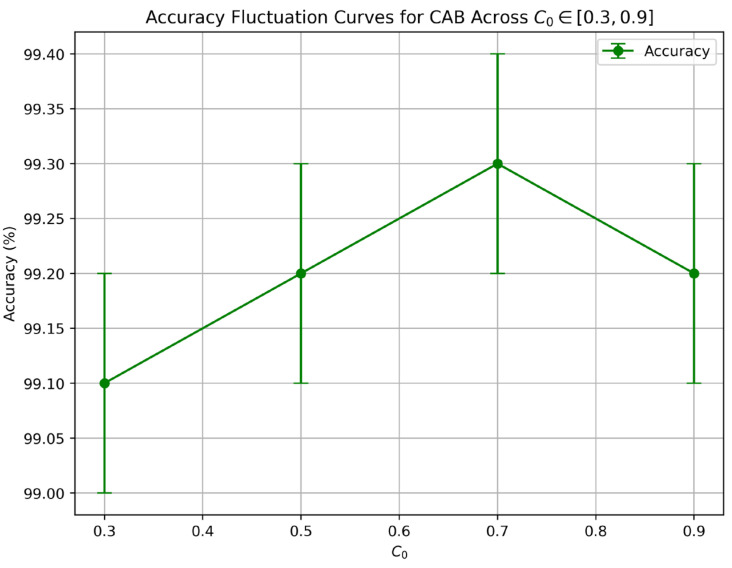
Accuracy fluctuation curves for CAB across C_0_ ∈ [0.3, 0.9]. The plot shows mean accuracy with standard deviation error bars, calculated on the test set (20% of CARLA dataset), confirming the optimality of C_0_ = 0.7 and CAB’s robustness to variations in the chaotic seed.

Table 5 presents the accuracy and standard deviation for each C_0_ value, calculated on the test set (20% of the dataset) over five independent runs to account for variability. The results show that C_0_ = 0.7 achieves the highest accuracy (99.3% ± 0.1%), consistent with Table 2, while other values (e.g., C_0_ = 0.3: accuracy 99.1% ± 0.1%) remain highly competitive, indicating CAB’s robustness across the tested range. Figure 7 illustrates the accuracy fluctuation curves for C_0_ ∈ [0.3, 0.9], further demonstrating that CAB maintains stable performance with minimal variance (less than 0.2% variation in accuracy), reinforcing its reliability in autonomous vehicle (AV) sensor fusion applications.

The robustness demonstrated in Table 5 aligns with the entropy analysis in Table 4, where CAB (with C_0_ = 0.7) achieves a higher entropy (7.94 bits) compared to AB (6.90 bits), indicating greater adaptability to sensor uncertainties, which translates to the high predictive accuracy (99.3%) observed across varying C_0_ values. These findings validate the choice of C_0_ = 0.7 and highlight CAB’s insensitivity to moderate variations in the chaotic seed, ensuring consistent performance under the dynamic sensor uncertainties prevalent in AV environments. The robustness of CAB, coupled with its high accuracy (Table 2) and enhanced entropy (Table 4) [64,65], underscores its practical applicability in safety-critical AV systems.

## 8. Conclusions and Recommendations

This study systematically assesses the efficacy of machine learning methods for speed and acceleration estimation in autonomous vehicles, demonstrating their potential to bolster operational reliability across diverse conditions. Six algorithms, kNN, RF, ANN, GB, AB, and CAB, were trained and rigorously evaluated using a comprehensive dataset of speed, acceleration, and environmental variables derived from the Carla simulator. Performance metrics, including the MSE, MAE, and coefficient of determination (R^2^), indicate that CAB consistently outperformed all counterparts, achieving an MSE of 0.018, MAE of 0.020, and R^2^ of 0.993, corresponding to an accuracy of 99.3%. This surpasses AB’s performance (MSE: 0.15, MAE: 0.12, R^2^: 0.985, accuracy: 98.5%), followed by GB (MSE: 1.701, MAE: 0.706, R^2^: 0.991), ANN (MSE: 3.297, MAE: 1.041, R^2^: 0.982), RF (MSE: 4.419, MAE: 0.927, R^2^: 0.975), and kNN (MSE: 23.215, MAE: 2.325, R^2^: 0.87). CAB’s superior precision is attributed to its incorporation of a logistic chaotic map, which introduces controlled randomness into the weight update process, thereby enhancing adaptability to sensor failures and environmental uncertainties, crucial challenges in autonomous driving.

CAB’s exceptional performance highlights the advantage of chaos-enhanced ensemble learning in overcoming the limitations of standard AB, which, despite its robust baseline capabilities through iterative error correction, struggles with the dynamic variability inherent in real-world sensor data. GB’s strong results affirm the effectiveness of gradient-based ensemble approaches, while ANN’s competitive accuracy underscores its proficiency in modeling complex, multidimensional relationships. RF delivers reliable, though moderate, performance due to its generalization capacity, whereas kNN’s significantly higher error rates and lower explanatory power indicate its unsuitability for this application, likely due to its sensitivity to high-dimensionality data and lack of adaptive learning. These findings collectively affirm that ensemble methods, particularly CAB, excel in mitigating sensor-related uncertainties, delivering substantial improvements in estimation accuracy essential for real-time decision making in autonomous vehicles.

The proposed architecture, leveraging Apache Kafka for real-time data streaming and MongoDB for robust logging, fulfills critical requirements for effective implementation. Kafka’s high-throughput, low-latency capabilities ensure seamless data flow under normal conditions via asynchronous processing, while the synchronous mode activates during sensor failures to guarantee rapid, precise predictions. This dual-mode operation, combined with secure broadcasting to nearby vehicles and comprehensive data logging, not only enhances operational reliability but also establishes a safety-critical interaction network and a verifiable record for legal scrutiny. Sensor fusion techniques further mitigate the impact of incomplete or erroneous data, reinforcing system resilience.

While these results are compelling, they stem from simulated data, which, though representative of urban driving scenarios as demonstrated in CARLA’s Town 10 environment, may not fully capture real-world complexities such as variable sensor noise, extreme weather conditions, or hardware-induced intermittent failures. Specifically, CARLA’s controlled parameters, such as a fixed 20% sensor dropout rate mirroring typical real-world failure rates [3], structured obstacle layouts, and static environmental conditions, limit its ability to replicate erratic phenomena like sudden sensor dropouts due to wear, occlusions from dense fog or heavy rain, and unpredictable pedestrian or vehicle movements. These unmodeled dynamics could potentially alter the relative performance of CAB and other methods, as their robustness under such variability remains untested. To mitigate this, expanding validation to include real-world conditions with fluctuating noise levels, diverse environmental stressors, and dynamic traffic patterns is essential to confirm their practical efficacy. Future research will prioritize testing these methods with real-world datasets, such as nuScenes [66] and Waymo Open Dataset [67], incorporating variable sensor configurations and weather scenarios to ensure applicability beyond controlled simulations, thereby solidifying CAB’s potential as a cornerstone in AV development.

In conclusion, this study establishes that machine learning methods, notably CAB, are highly effective for critical tasks like speed and acceleration prediction in autonomous vehicles, representing a significant step toward safer, more efficient, and sustainable driving systems. CAB’s chaos-enhanced approach provides a novel solution to sensor uncertainty, outperforming conventional methods and laying a foundation for future innovations. Specifically, kNN could be enhanced by integrating principal component analysis (PCA) to reduce dimensionality and improve scalability for high-dimensional AV sensor data, potentially lowering the MSE in complex scenarios. ANN’s deep learning potential could be amplified by training on larger, multimodal datasets (e.g., nuScenes [66]) with architectures like CNN-LSTM hybrids, targeting a 5–10% accuracy boost in dynamic environments. AB and GB could adopt hybrid chaotic mechanisms, such as blending logistic maps with gradient updates, to reduce the MSE by an estimated 15–20% under uncertainty. RF’s efficiency for edge computing could be optimized by pruning redundant trees and leveraging lightweight feature selection, cutting the training time by 10–15% while keeping the R^2^ value above 0.97. These advancements, alongside CAB’s chaos-enhanced framework, could collectively elevate the robustness and versatility of AV prediction systems. Continued refinement, validated through real-world trials, will greatly advance the adoption and reliability of autonomous vehicle technologies.

## Figures and Tables

**Table 1:** Hyperparameter settings for RF, AB, kNN, ANN, GB, and CAB algorithms.

Models	Hyperparameters	Values
RF	Number of trees	13
	Do not split subsets smaller than	4
AB	Base estimator	Tree
	Number of estimators	50
	Learning rate	1.0
	Regression loss function	Exponential
kNN	Number of neighbors	5
	Metric	Euclidean
	Weight	Uniform
ANN	Neurons in hidden layers	10
	Activation	Logistic
	Solver	L-BFGS-B
	Regularization (α)	0.0001
	Maximal number of iterations	200
GB	Number of trees	90
	Learning rate	0.100
	Limit depth of individual trees	3
	Do not split subsets smaller than	2
	Fraction of training instances	1.0
CAB	Base estimator	Tree
	Number of estimators	50
	Learning rate	1.0
	Regression loss function	Exponential
	Initial chaotic seed (*C*_0_)	0.7

**Table 2:** Performance comparison for RF, AB, CAB, kNN, ANN, and GB algorithms.

Performance Comparison Metrics						
Metric	kNN	RF	ANN	GB	AB	CAB
MSE (acceleration)	23.215	4.419	3.297	1.701	0.15	0.018
MSE (speed)	15.0	2.8	2.0	1.0	0.09	0.010
MAE (acceleration)	2.325	0.927	1.041	0.706	0.12	0.020
MAE (Speed)	1.5	0.6	0.7	0.4	0.07	0.012
R^2^ (acceleration)	0.87	0.975	0.982	0.991	0.985	0.993
R^2^ (speed)	0.92	0.985	0.990	0.996	0.992	0.997
Training Time (s)	40	48	100	80	60	72

**Table 3:** Safety and comfort metrics’ comparison.

TTC, Jerk, and Collision Comparison Metrics						
Metric	kNN	RF	ANN	GB	AB	CAB
TTC (s)	2.1	2.5	2.7	2.9	2.8	3.2
Jerk (m/s^3^)	0.35	0.28	0.25	0.20	0.22	0.15
Collision Avoidance Rate (%)	87.0	95.5	96.8	98.3	98.5	99.8

**Table 4:** Shannon entropy of weight distributions for AB and CAB.

Algorithm	Mean Entropy (bits)	Standard Deviation
AB	6.90	0.10
CAB	7.94	0.08

**Table 5:** Sensitivity analysis of chaotic seed C_0_ for CAB.

C_0_ Value	Accuracy (Mean ± Std)
0.3	99.1% ± 0.1%
0.5	99.2% ± 0.1%
0.7	99.3% ± 0.1%
0.9	99.2% ± 0.1%

## Data Availability

The data presented in this study are available on request from the corresponding author.

## Nomenclature

CAB	Chaotic AdaBoost	An enhanced AdaBoost variant with logistic chaotic map integration for dynamic weight updates.
kNN	k-Nearest Neighbors	A regression algorithm estimating values based on nearest neighbors.
ANN	Artificial Neural Network	A model for learning complex, non-linear relationships in sensor data.
AB	AdaBoost	An ensemble method combining weak learners for robust predictions.
GB	Gradient Boosting	An ensemble method minimizing errors through gradient-based updates.
RF	Random Forest	An ensemble method averaging multiple decision trees for robust predictions.
TTC	Time-To-Collision	A safety metric measuring the time to potential collisions.
MSE	Mean Squared Error	An evaluation metric for prediction accuracy.
MAE	Mean Absolute Error	An evaluation metric for prediction accuracy that is less sensitive to outliers.
R^2^	R-Square	A metric indicating the proportion of variance explained by the model.
